# Knowledge, Attitude, and Practices on Drug-Resistant Tuberculosis Infection Control in Nepal: A Cross-Sectional Study

**DOI:** 10.1155/2021/6615180

**Published:** 2021-03-01

**Authors:** Sailesh Kumar Shrestha, Ratna Bahadur Bhattarai, Lok Raj Joshi, Nilaramba Adhikari, Suvesh Kumar Shrestha, Rajendra Basnet, Kedar Narsingh K. C.

**Affiliations:** ^1^National Academy of Medical Sciences, Bir Hospital, Kathmandu 44600, Nepal; ^2^National Tuberculosis Control Center, Bhaktapur 44800, Nepal; ^3^Central Department of Public Health, Institute of Medicine, Kathmandu 44600, Nepal; ^4^Save the Children/Global Fund, Kathmandu 44600, Nepal

## Abstract

Drug-resistant tuberculosis (DR-TB) transmission is an important problem, particularly in low-income settings. This study is aimed at assessing the knowledge, attitude, and practices of DR-TB infection control among the healthcare workers under the National Tuberculosis Control Program in Nepal. In this cross-sectional study, we studied the healthcare workers from all the 11 functioning DR-TB treatment centers across Nepal in March 2018. Through face-to-face interviews, trained data collectors collected data on the characteristics of healthcare workers, their self-reported knowledge, attitude, and practice on DR-TB infection control. We entered the data in Microsoft Excel and analyzed in the R statistical software. We assigned a score of one to the correct response and zero to the incorrect or no response and calculated a composite score in each of the knowledge, attitude, and practice domains. We ascertained the healthcare workers as having good knowledge, appropriate attitude, and optimal practices when the composite score was ≥50%. We summarized the numerical variables with median (interquartile range (IQR)) and the categorical variables with proportions. We ran appropriate correlation tests to identify relationships between knowledge, attitude, and practice scores. We regarded a *p* value of <0.05 as significant. A total of 95 out of 102 healthcare workers responded. There were 46 male respondents. The median age was 33 years (IQR 26-42). Most of them (53, 55.79%) were midlevel paramedics. We found 91 (95.79%) respondents had good knowledge, 49 (51.58%) had an appropriate attitude, and 35 (36.84%) had optimal practices on DR-TB infection control. We found a statistically significant positive correlation between attitude and practice scores (*ρ* = 0.37, *p* ≤ 0.001). The healthcare workers at the DR-TB treatment centers in Nepal have good knowledge of DR-TB infection control, but it did not translate into an appropriate attitude or optimal practices.

## 1. Background

Drug-resistant tuberculosis (DR-TB), a form of tuberculosis (TB) resistant to one or more antitubercular drugs, has emerged as a major public health challenge. Globally, in 2018 alone, there were an estimated 484000 new cases of TB caused by the bacilli resistant to Rifampicin (RR TB), out of which 78% were additionally resistant to Isoniazid (Multidrug Resistant TB (MDR-TB)). Furthermore, the bacilli infecting 6.2% of the MDR-TB cases were additionally resistant to at least a Fluoroquinolone and a second line injectable antitubercular agent (Extensively Drug-Resistant Tuberculosis (XDR-TB)). More than 90% of these cases are from 30 countries that belong to low or middle-income settings [1]. In Nepal, of the 1400 estimated cases of RR/MDR-TB, 635 cases of MDR-TB were notified to the National TB Control Program (NTP) in 2018 [2].

Until recently, the provision of effective first-line treatment was hoped to prevent the emergence of DR-TB. However, growing evidence suggests that person-to-person transmission, not just inadequate treatment, is driving the spread of DR-TB. A dynamic transmission modeling analysis using national tuberculosis notification data estimated that person-to-person transmission rather than *de novo* acquisition accounts for a median of 95.9% of all incident MDR-TB and 61.3% of incident MDR-TB in previously treated individuals [3]. In a prospective study of 404 patients with XDR-TB in South Africa, investigators combined genotyping methods with social-network and epidemiologic analysis and found that at least 69% of the cases of XDR-TB were attributable to transmission [4]. In another study on 324 patients with MDR-TB in China, investigators combined traditional genotyping, whole-genome sequencing, and epidemiological investigation and found that transmission of MDR strains accounted for 73% of cases of MDR-TB [5].

Healthcare centers represent one of the important sites where DR-TB transmission occurs, particularly in settings where the infection control measures are often poor, and the burden of TB is high [6]. A study from Moldova, a high MDR-TB burden country, evaluated 67 patients with MDR-TB being treated as in-patients and followed them up. It was found that 50 patients (74.6%) had a strain in the follow-up that was different from that of the baseline suggesting a high proportion of MDR-TB possibly transmitted during in-patient treatment [7].

Standard guidelines on TB infection control could help minimize healthcare-based DR-TB transmission and should form an indispensable part of DR-TB care. Optimal utilization and implementation of such guidelines could be facilitated by the information on the knowledge gaps, attitudes, and practices on TB infection control of the healthcare workers. However, in many resource-limited settings, TB infection control measures are absent or suboptimal. In Nepal, dedicated national policy and resources for TB infection control are lacking, and a few studies have assessed healthcare workers' knowledge, attitudes, and practices on TB infection control. A study among 190 healthcare workers from 28 TB treatment facilities in Nepal found that the overall knowledge and practices of healthcare workers on TB infection control were not satisfactory [8]. This study, however, was limited to only the urban centers, and the knowledge, attitudes, and practices questionnaire did not extend to include the issues specific to DR-TB. Such information could help in the tailored implementation of the guidelines and thus, more effective take-up of the recommendations. In this study, we aimed to assess the knowledge, attitude, and practices of DR-TB infection control among the healthcare workers under the National Tuberculosis Control Program in Nepal.

## 2. Materials and Methods

### 2.1. Study Settings and Participants

We conducted this cross-sectional study among the healthcare workers at the DR-TB treatment centers in Nepal in March 2018. DR-TB treatment in Nepal is ambulatory care provided through DR-TB treatment centers under the NTP. Depending upon the volume of patients and their geographic distributions, the NTP identifies different health centers across the country to introduce the DR-TB treatment services. After orientation training of the healthcare workers on DR-TB care and the provision of required logistics, these centers then start functioning as the DR-TB treatment centers. They evaluate the presumptive cases of DR-TB in close collaboration with the national TB reference laboratories, provide the DR-TB treatment to the diagnosed patients, and monitor them as per the national guidelines. All the patients diagnosed with DR-TB are enrolled at one of these centers. There were 18 DR-TB treatment centers across the country during the data collection period, of which we included 11 centers that had patients enrolled and were receiving treatment. There were 102 healthcare workers involved in the care of the patients with DR-TB at these 11 centers, and we included all of them in our study.

### 2.2. Data Collection

In a one-day training session, we trained six paramedics on the details of selecting study participants, the process of obtaining informed consent, and data collection using the structured data collection tool. Because no standardized data collection instrument was available to evaluate the knowledge, attitude, and practice in patients with DR-TB, we developed a tool based on earlier studies [9, 10]. We pretested the tool for any problems in understanding or interpreting the questionnaire and incorporated the feedback in the final version. The pretesting was done in 10 healthcare workers at the TB clinic of the National TB Control Center who did not form a part of the actual study sample in the data collection process of our study. The data collection tool consisted of four parts. The first part collected information on the study participants' demographic and other characteristics related to the delivery of DR-TB services (current position at work, work experience in general and specific to DR-TB care, training received on TB infection control, and whether the study participants had encountered colleagues with TB or DR-TB). The midlevel paramedics referred to the health workers working as community health workers or health assistants and required vocational education at upper secondary or postsecondary, nontertiary levels. However, their actual educational qualification, as entered separately in the variable–education, may be of higher level. We classified the education level according to the International Standard Classification of Education [11]. The second part contained a questionnaire on the knowledge which collected information on the study participants' knowledge on the risk of getting infected with DR-TB and the administrative, environmental, and personal protective measures of TB infection control. The responses were recorded as true, false, and do not know. The third part contained a questionnaire on attitude which collected information on the study participants' attitude towards DR-TB and measures of TB infection control. The responses were recorded as agree, disagree, and no comments. The fourth part contained questions on the study participants' self-reported practices on infection control measures while encountering the patients with DR-TB. The responses were recorded as yes, no, and do not know.

### 2.3. Data Analysis

We entered the data in Microsoft Excel (Office 365, Microsoft Corporation, Washington, United States) and analyzed in the R statistical software (R Core Team (2020). R: A language and environment for statistical computing. R Foundation for Statistical Computing, Vienna, Austria. https://www.R-project.org/). We summarized the numerical variables with median and interquartile range (IQR) and the categorical variables with proportions. We assigned a score of one to the correct response and zero to the incorrect or no response and calculated a composite score in each of the knowledge, attitude, and practice domains. We ascertained the healthcare workers as having good knowledge, appropriate attitude, and optimal practices when the composite score in each of the domains was at least 50%. We ran appropriate correlation tests to identify relationships between knowledge, attitude, and practice scores. A *p* value of <0.05 was considered significant.

### 2.4. Ethical Considerations

We obtained ethical approval for the study from Nepal Health Research Council (registration number 109/2018, approval reference number 2328/2018). The trained data collectors obtained written informed consent from the participants for participation in the study.

## 3. Results

Of the 102 healthcare workers from 11 DR-TB treatment centers, 95 participated in the study (response rate—93.1%). Altogether, there were 14 missing values across five variables. We assumed that the missing values were missing completely at random and analyzed the remaining data with complete case analysis. The median age was 33 (IQR 26-42) years, and there were 49 (51.58%) female respondents. Most of them (53, 55.96%) were midlevel paramedics, and 44 (46.32%) study participants had received postsecondary nontertiary vocational education (Table 1).

The median work experience was 10 (IQR 3–18) years and that in DR TB was 2 (IQR 1–6) years. Eighty-eight (92.63%) study participants were providing health services to the patients in addition to the patients with DR-TB. Seventy-five (78.95%) study participants reported to have encountered colleagues with TB, and 38 participants (40.00%) reported to have encountered colleagues with DR-TB. The majority (71, 74.74%) of the study participants reported that they had received training on DR-TB infection control.

**Figure 1:**
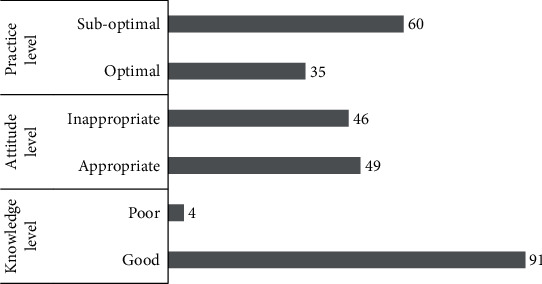
Number of study participants with the knowledge, attitude, and practice levels.

**Figure 2:**
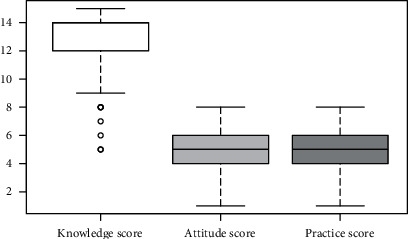
Boxplot of total knowledge, attitude, and practice scores.

The majority of the study participants responded correctly to the knowledge questions except for the questions on administrative control measures and use of surgical masks by the patients—close to a third of the study participants failed to appreciate the administrative control and use of surgical masks by the DR-TB patients as the crucial measures of TB infection control (Table 2). Yet, a large proportion of healthcare workers (91, 95.79%) were found to have a good knowledge of DR-TB infection control with the median knowledge score of 14 (IQR 12-14) (Figures 1 and 2).

Just over half of our study participants (49, 51.58%) were found to have an appropriate attitude on DR-TB infection control with a median attitude score of 5 (IQR 4-6) (Figures 1 and 2). More than half of the study participants did not think they needed to use N95 respirators while taking care of DR-TB patients, and close to half of the study participants undermined the role of administrative and environmental infection control measures in DR-TB infection control (Table 3). Many study participants thought that their workplaces neither had adequate resources for DR-TB infection control nor were concerned over the risk of DR-TB transmission to the healthcare workers. Over one-third of the study participants felt that DR-TB cannot be cured, and taking infection control precautions will offer a little help to the healthcare workers since they may have already been infected with DR-TB. However, most of them believed that they had been well trained in TB infection control and showed interest in getting tested for TB periodically.

Overall, only over a third of our study participants (35, 36.84%) were found to have optimal practices of DR-TB infection control with the median practice score of 5 (IQR 4-7) (Figures 1 and 2). Around a third of study participants reported poor practices on administrative measures of infection control, the lack of infection control program along with an infection control committee or a focal person in their workplace, the lack of triaging actively coughing patients to expedite the care they receive in the workplace, and the lack of an appropriate area for sputum collection (Table 4). The resources available for infection control were reportedly inadequate as well. Nearly half of the study participants reported that the ventilation in the patient consultation rooms and laboratories was suboptimal, and exhaust ventilation and ultraviolet radiation were almost nonexistent. Though a significant proportion of study participants reported that they encouraged patients to use surgical masks, less than a quarter of them used N95 respirators themselves when caring for the patients with DR-TB.

All the three variable scores (knowledge, attitude, and practice) were not normally distributed, and therefore, we ran Spearman's rank correlations. We found a significant positive correlation between attitude score and practice score (*ρ* = 0.37, *p* ≤ 0.001) but not between knowledge and attitude scores (*ρ* = −0.09, *p* = 0.38) nor between knowledge and practice scores (*ρ* = −0.18, *p* = 0.079).

## 4. Discussion

This study is aimed at assessing the knowledge, attitude, and practices of DR-TB infection control among the healthcare workers under the NTP in Nepal and is the first such study in Nepal to be conducted nationwide. We found that most of the study participants had a good knowledge of DR-TB infection control. However, the knowledge score did not correlate with the attitude or practice scores. Only half of the healthcare workers studied had an appropriate attitude, and just over a third had optimal practices on DR-TB infection control. However, attitude score significantly correlated with the practice score.

The NTP in Nepal has been gradually expanding its DR-TB treatment services across the country based on the number of patients reported, their geographic distributions, and available resources. Before introducing the DR-TB treatment services in any health facility, the NTP conducts a five-day-long orientation training on DR-TB care to all the healthcare workers involved. Thereafter, once every three to five years, a three-day-long refresher training is provided as a part of the continuing professional development program. Each of these training programs includes a session on infection control and is, so far, the only formal DR-TB infection control training session available within Nepal NTP. Most of the study participants reported that they had received training on TB infection control. And with the median work experience in DR-TB care of 2 years, most of the study participants had received this training quite recently. These training sessions might have led to a good knowledge of TB infection control in our study participants. In addition, the educational qualification of the study participants could have also contributed to the higher knowledge of DR-TB infection control.

The knowledge scores on DR-TB infection control reported in the earlier studies vary widely. In a national survey of 24 DR-TB facilities involving 499 healthcare workers in South Africa, the average number of correct responses was 3.1 out of 10 knowledge questions [10]. In another study on 377 healthcare workers from two regional referral hospitals in Ethiopia, it was found that the mean knowledge score was seven out of the total possible knowledge score of 10 [12]. Such variation in the knowledge levels reflects the variation in the study settings, including whether the healthcare workers studied were from DR-TB specific facilities as well as the study population, including their educational qualifications, training on TB infection control, and work experience. In both these studies, however, a higher level of clinical training and attending infection control training was associated with significantly higher knowledge scores [10, 12]. Though not conducted on the healthcare workers working exclusively on DR-TB facilities, an earlier study from Nepal has also reported a significant association of education level and training on TB with the respondents having a good knowledge of TB infection control [8]. These findings underscore the importance of training opportunities for the healthcare workers in TB infection control, including engaging the healthcare workers in short-term refresher training and encouraging continuous learning. Such training sessions in the future should reinforce the importance of administrative control as a key strategy for DR-TB infection control. Also, it should be emphasized that the use of surgical masks by the patients is an important measure for source control leading to a fewer number of tubercular bacilli being released into the surrounding.

Our finding that close to half of the study participants had an inappropriate attitude on DR-TB infection control including in the key areas like the role of administrative and environmental infection control measures in DR-TB infection control and the use of N95 respirators by healthcare workers while taking care of DR-TB patients is concerning. These issues put the healthcare workers at an increased risk of acquiring DR-TB infection themselves. Reported attitudes on DR-TB infection control vary between the different studies. In the study in South Africa, nearly one-third of participating healthcare workers from DR-TB facilities reported not using N95 respirators during patient care [10]. On the other hand, almost all the study participants from an earlier study in Nepal agreed that they should wear respirators while caring for TB patients. A higher proportion of them seemed to be concerned about getting infected with TB in comparison to our study participants (Table 3) [8]. Various factors help shape such attitudes on infection control including the educational qualification, whether they have received training specific to TB infection control, and the level of knowledge on infection control [8, 10].

Fewer healthcare workers in our study had optimal practices of DR-TB infection control, particularly on their practices on administrative infection control measures and the use of N95 respirators while caring for the patients with DR-TB. In the earlier studies, the use of N95 respirators during the care of the patients with TB varied from 10% to 39.7% likely reflecting the underlying differences in the study population and their educational qualification as well as the training on TB infection control [8, 10, 12]. While the provision of adequate resources for infection control, including exhaust ventilation and ultraviolet radiation, should be a goal as well, it is the implementation and adherence to relatively cheaper yet more effective measures such as triage of actively coughing patients, reinforcing cough etiquette and a separate well ventilated sputum collection area that should take the precedence.

In our study, a higher score on attitude was associated with a higher score on TB infection control practices, but a higher knowledge score was not associated with higher attitude or practice scores. These findings suggest that good knowledge, though a prerequisite, does not necessarily translate into an appropriate attitude or optimal practices of healthcare workers on DR-TB infection control. The existing training by the NTP has helped the study participants attain a good knowledge on TB infection control, but it has had a little positive impact on the attitude and practices among them. Similar observations have been reported in earlier studies as well. A review on the role of education in infection prevention and control found that while training may improve knowledge, it does not always effectively or sustainably change practice [13]. A systematic review on the barriers and facilitators of tuberculosis infection prevention and control in low- and middle-income countries has also failed to find robust evidence that education improves practice and reduces infection rates in the long run [14]. Two important considerations apply in our study—the current NTP training focuses on the clinical management of the patients with DR-TB failing to highlight the issues on DR-TB infection control and consists of didactic lectures failing to highlight the much needed practical aspects. Training programs that are specifically designed and tailored to educate the healthcare workers on DR-TB infection control need to be implemented that could help build up more appropriate attitudes and improve the DR-TB infection control practices. The need for training sessions specific to TB infection control was also recognized by the earlier study from Nepal [8]. These training programs should help the health workers obtain the skills necessary for optimal infection control practices, for example, the appropriate use of N95 respirators. Besides, the way the NTP delivers the TB infection control training sessions needs to be redesigned as well. We recommend that Nepal NTP take a different approach, for example, using strategies like behavior change communication. This approach is different from the traditional approach of providing information and expecting this to result in changed behavior. Rather, it envisions communications as a horizontal process so that both senders and receivers of the information can take on interchangeable roles through focus group discussions and other methods to reach a mutual understanding and collective decision-making. At least a proportion of health workers, particularly those with good knowledge, could also be motivated to adhere to the TB infection control guidelines with the provision of regular supervision and of penalties/loss of benefits or of rewards depending on whether or not they adhere to the TB infection control recommendations. A study from Russia, which has found loss of benefit payments as an important motivator affecting the TB infection control practices among Russian health workers, supports this hypothesis [15]. This should be reinforced with other measures including ensuring the availability of necessary resources, for example, N95 respirators, creating a supportive environment during supervision visits and interventions to help minimize the stigma surrounding DR-TB. Collectively, these approaches constitute behavior change interventions that are found to be effective to change the practice of health workers to bring about sustained behavioral changes [16].

Our study has some limitations. The questionnaire used in this study has not been assessed for reliability and validity thus limiting the scope of the inferences and conclusions made based on the responses obtained. Besides, the respondents on whom we pretested the study questionnaire belonged to a single center which may not have adequately captured the variation across the study population. Due to time and resource constraints, we recorded TB infection control practices that were self-reported by the study participants and not observed by the data collectors or the investigators. These self-reported practices could differ from actual practices [10]. Therefore, for future studies, we recommend that infection control practices be directly observed by the data collectors and/or investigators. We did not assess the factors that could predict the good knowledge, appropriate attitude, or optimal practices on DR-TB infection control. We recommend that in subsequent studies, such factors and their impact be studied as well, which could help focus the interventions on the key issues on DR-TB infection control.

## 5. Conclusion

This study has shown that the healthcare workers at the DR-TB treatment facilities in Nepal have good knowledge of DR-TB infection control. However, it did not translate into the appropriate attitude or optimal practices. This suggests the need to adopt focused TB infection control training for healthcare workers using strategies like behavior change communication, along with several other behavioral change interventions that could bring about positive changes in their attitude and practices.

## Figures and Tables

**Table 1:** Characteristics of study participants.

Characteristics	Frequency (*n*)	Percentage (%)
Age groups		
16-30 years	42	44.21
31-45 years	39	41.05
46-60 years	14	14.74
Sex		
Female	49	51.58
Male	46	48.42
Education		
Postsecondary, nontertiary, vocational education	44	46.32
Bachelors	28	29.47
Upper secondary vocational	13	13.68
Masters	10	10.53
Position		
Midlevel paramedic	53	55.79
Laboratory personnel	17	17.89
Nurse	13	13.68
Program manager	6	6.32
Doctor	3	3.16
Radiographer	3	3.16
Health facility type		
Government	55	57.89
Nongovernment	40	42.11
Work experience as a healthcare worker		
1-10 years	50	53.76
11-20 years	26	27.96
21-30 years	13	13.98
31-40 years	4	4.30
Work experience in drug-resistant tuberculosis		
5 years or less	64	73.56
6-10 years	11	12.64
11-20 years	12	13.79

**Table 2:** Knowledge questions on drug-resistant tuberculosis infection control and the responses from the study participants.

Knowledge questions	True		False		Do not know		Total responses
	*n*	%	*n*	%	*n*	%	
Drug-resistant tuberculosis is an infectious disease.	85	91%	—	—	8	9%	93
Drug-resistant tuberculosis is more infectious than drug-sensitive tuberculosis.	76	80%	10	11%	9	9%	95
Healthcare workers have a higher risk of acquiring tuberculosis than the general population.	89	94%	5	5%	1	1%	95
The risk of tuberculosis to healthcare workers can be minimized by observing appropriate infection control measures.	83	87%	5	5%	7	7%	95
Healthcare facilities should implement an infection control program.	88	94%	5	5%	1	1%	94
Administrative measures are the most effective tuberculosis infection control measures.	57	60%	28	29%	10	11%	95
The actively coughing patients should be identified, and prompt care should be provided to minimize the time spent at healthcare facilities.	87	92%	—	—	8	8%	95
The actively coughing patients should be encouraged to cover their mouth and nose while coughing or sneezing.	87	92%	—	—	8	8%	95
Patients with tuberculosis and their close contacts should be educated on the measures of tuberculosis infection prevention.	86	91%	1	1%	8	8%	95
The waiting area and sputum collection area should be in an open and well-ventilated space.	85	89%	10	11%	—	—	95
The health facility should be renovated in such a way that patient consultation rooms and laboratories are well ventilated.	84	88%	3	3%	8	8%	95
The use of mechanical ventilation and ultraviolet gamma irradiation in health facilities helps to minimize tuberculosis transmission.	86	91%	—	—	9	9%	95
Surgical masks are as good as N95 masks in preventing tuberculosis transmission.	22	23%	70	74%	3	3%	95
Healthcare workers should wear N95 masks.	92	97%	3	3%	—	—	95
Patients should wear surgical masks.	64	67%	29	31%	2	2%	95
Healthcare workers should be screened for tuberculosis periodically.	89	94%	4	4%	2	2%	95

**Table 3:** Attitude questions on drug-resistant tuberculosis infection control and the responses from the study participants.

Attitude questions	Agree		Disagree		No comments		Total responses
	*n*	%	*n*	%	*n*	%	
I am worried that I may contract drug-resistant tuberculosis.	18	19%	28	29%	49	52%	95
Drug-resistant tuberculosis cannot be cured.	32	34%	60	63%	3	3%	95
My workplace is concerned about the risk of drug-resistant tuberculosis transmission to its staff.	45	47%	40	42%	10	11%	95
My workplace has provisioned adequate resources to minimize the risk of drug-resistant tuberculosis transmission to its staff.	32	34%	59	62%	4	4%	95
I have been well trained in tuberculosis infection control.	50	53%	36	38%	9	9%	95
Healthcare workers have already been infected with tuberculosis, so taking infection control measures will not help.	37	39%	57	60%	1	1%	95
The administrative measures of tuberculosis infection control minimize the exposure to tubercular bacilli.	46	48%	45	47%	4	4%	95
The environmental measures of tuberculosis infection control minimize the number of tubercular bacilli in the environment.	42	45%	44	47%	8	9%	94
The healthcare workers taking care of the patients with drug-resistant tuberculosis must wear N95 masks.	39	41%	56	59%	—	—	95
I would like to be tested for tuberculosis periodically.	56	59%	39	41%	—	—	95

**Table 4:** Practice questions on drug-resistant tuberculosis infection control and the responses from the study participants.

Practice questions	Yes		No		Do not know		Total responses
	*n*	%	*n*	%	*n*	%	
My workplace has a tuberculosis infection control committee.	36	38%	37	39%	22	23%	95
There is a tuberculosis infection control focal person ascertained in my workplace.	42	44%	32	34%	21	22%	95
There is a tuberculosis infection control program implemented in my workplace.	59	62%	26	27%	10	11%	95
Adequate resources have been allocated for tuberculosis infection control in my workplace.	50	53%	38	40%	7	7%	95
Healthcare workers have been tested for latent tuberculosis infection.	31	33%	50	53%	14	15%	95
The actively coughing patients are identified and provided prompt care to minimize the time spent in my workplace.	61	64%	31	33%	3	3%	95
A separate and open area has been designated as a sputum collection area in my workplace.	55	58%	35	37%	5	5%	95
There are signs of cough etiquette displayed in my workplace.	37	39%	56	59%	2	2%	95
The patient consultation rooms and laboratory in my workplace are well ventilated.	52	55%	41	43%	2	2%	95
There is exhaust ventilation available in my workplace.	—	—	87	92%	8	8%	95
There is ultraviolet irradiation available in my workplace.	—	—	91	96%	4	4%	95
I use an N95 mask most of the time.	22	23%	73	77%	—	—	95
I encourage patients to use surgical masks.	85	89%	10	11%	—	—	95

## Data Availability

The data used to support the findings of this study are available from the corresponding author upon request.
